# Immune-Epithelial Cross Talk in Regeneration and Repair

**DOI:** 10.1146/annurev-immunol-101721-062818

**Published:** 2023-01-25

**Authors:** Laure Guenin-Mace, Piotr Konieczny, Shruti Naik

**Affiliations:** 1 Department of Pathology, NYU Langone Health, New York, NY, USA; 2 Immunobiology and Therapy Unit, INSERM U1224, Institut Pasteur, Paris, France; 3 Department of Medicine, Ronald O. Perelman Department of Dermatology, and Perlmutter Cancer Center, NYU Langone Health, New York, NY, USA

**Keywords:** repair, regeneration, immunity, inflammatory disease, epithelium, stem cells

## Abstract

The epithelial tissues that line our body, such as the skin and gut, have remarkable regenerative prowess and continually renew throughout our lifetimes. Owing to their barrier function, these tissues have also evolved sophisticated repair mechanisms to swiftly heal and limit the penetration of harmful agents following injury. Researchers now appreciate that epithelial regeneration and repair are not autonomous processes but rely on a dynamic cross talk with immunity. A wealth of clinical and experimental data point to the functional coupling of reparative and inflammatory responses as two sides of the same coin. Here we bring to the fore the immunological signals that underlie homeostatic epithelial regeneration and restitution following damage. We review our current understanding of how immune cells contribute to distinct phases of repair. When unchecked, immune-mediated repair programs are co-opted to fuel epithelial pathologies such as cancer, psoriasis, and inflammatory bowel diseases. Thus, understanding the reparative functions of immunity may advance therapeutic innovation in regenerative medicine and epithelial inflammatory diseases.

## INTRODUCTION

Tissue damage is central to the pathology of injury, infection, autoimmunity, and cancer. These assaults result from both external threats (e.g., pathogens, noxious agents, trauma) and internal perturbations (e.g., mutations, unchecked immune activity) and profoundly disrupt tissue homeostasis. Damage can be discrete in the case of localized injury such as a cut or scrape or extend across the entire organ and compromise function. Ultimately, persistent damage or a failure to engage effective repair mechanisms of vital organs can be catastrophic and result in death. For instance, respiratory failure and the ensuing fatality of the COVID-19 pandemic were traced to ineffective engagement of lung epithelial repair programs (1, 2). Thus, in addition to pathogen control, engaging repair mechanisms is essential for mitigating tissue pathology and restoring organ structure and function. Indeed, an astounding 45% of all deaths in the Western world can be attributed to reparative failures and fibrosis (3).

While immune cells are often the purveyors of damage and destruction, they also possess remarkable healing powers. Clinical observations of impaired repair in immunocompromised individuals underscore a central role for immunity in wound healing (4). The presence of immune cells at the site of injury was first noted by Elie Metchnikoff over a century ago (5). Metchnikoff famously observed phagocytes (macrophages) after poking starfish larvae with a rose thorn. Extending Metchnikoff’s findings to mammalian systems, early studies examining the cellular contexture of rabbit ear wounds also noted an enrichment of macrophages and monocytes (6). In the 1970s and 1980s, researchers used depleting antimacrophage serum to examine the function of macrophages and monocytes in repair (7-9). These landmark studies identified delays in dermal fibroblast responses, vascular responses, and collagen synthesis. Importantly, these findings shifted views of macrophages from merely big eaters that phagocytosed microbial and cellular debris to cells that provided vital signals that direct tissue growth. Further insights into the role of macrophages in wound repair were inferred from studies of worm and parasite infections, which led to a dichotomous view of proinflammatory M1 and pro-repair M2 macrophages (10). The field has since embraced a more nuanced understanding of the macrophage state, as the mechanistic underpinnings of the initial observations of macrophages and other innate cells in repair have unfolded (11, 12).

In the last 20 years, researchers have uncovered the remarkable complexity of the adaptive immune system, including numerous subsets of innate-line and adaptive lymphocytes that permanently reside in tissues and actively converse with the surrounding stroma (13). Whereas the field of wound repair has historically focused on innate immune cells, a flurry of recent literature points to a role for adaptive immune cells in dialoguing with the epithelia at steady state and in orchestrating epithelial repair following injury. As our understanding of noncanonical tissue regulatory functions of adaptive immunity deepens, there is a growing appreciation for the functional coupling of reparative and inflammatory responses as two sides of the same coin.

Here we provide a comprehensive overview of the immunological mechanisms underlying homeostatic epithelial regeneration and tissue repair (Tables 1, 2). We focus on the skin and small intestine epithelia, two tissues that continually regenerate in health and are prone to injury, infections, and inflammation that cause damage. Thus, both the skin and gut evolved sophisticated repair mechanisms that are geared to rapidly restore epithelial barrier function. We first discuss the homeostatic dialogue between immune cells and the epithelium as it relates to barrier maintenance. We then delve into the repair mechanisms that are engaged upon barrier breach. These occur in distinct but temporally overlapping phases and are heavily influenced by immunity. In particular, we focus on re-epithelialization following injury and also touch upon the conversations that take place between immune cells and other tissue constituents (e.g., endothelium, neurons, mesenchyme) to support this process. When unchecked, epithelial repair programs are co-opted in autoimmune conditions and cancers. Finally, we discuss the pathological repair mechanisms driving these diseases and the tremendous potential of leveraging the immune-epithelial cross talk therapeutically. As knowledge of immune-epithelial conversations expands, so too will opportunities emerge for reparative therapies and treatments for chronic inflammatory diseases that propel regenerative medicine to new heights.

## IMMUNE-EPITHELIAL CROSS TALK IN HOMEOSTATIC REGENERATION

**Figure 1:**
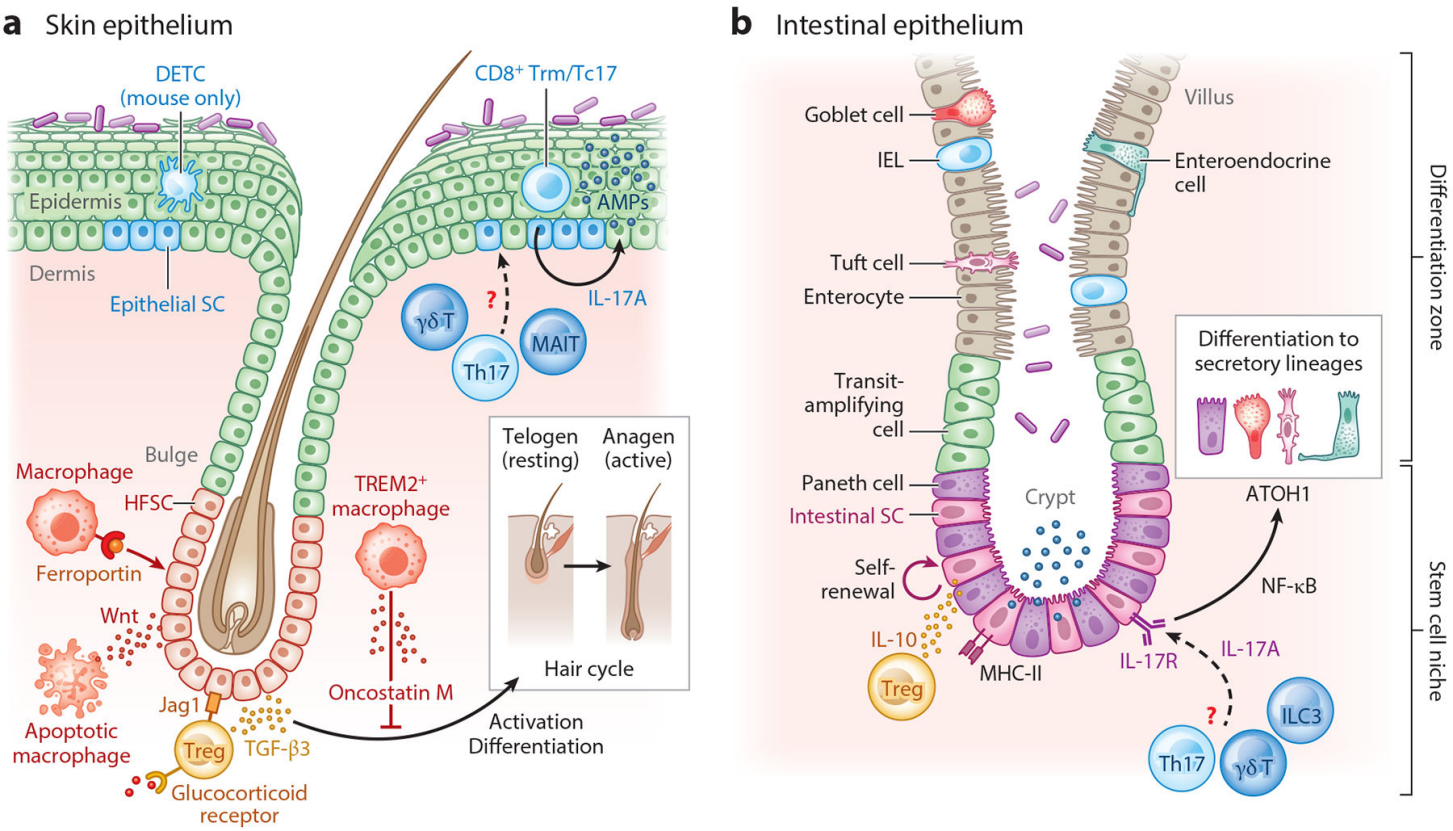
Immune-epithelial cross talk in homeostatic regeneration of the (*a*) skin and (*b*) gut. (*a*) HFSCs reside in the hair follicle bulge and fuel the hair cycle, while epidermal stem and progenitor cells differentiate from the basal (lowermost) layer to sustain homeostatic epidermal turnover. Perifollicular macrophages and Tregs dynamically modulate HFSC behavior. TREM2^+^ macrophages secrete oncostatin M to maintain HFSC quiescence and promote telogen, or the resting phase of the hair cycle. Macrophages supply iron to HFSCs via the transmembrane receptor ferroportin, and apoptotic macrophages release Wnts to activate HFSCs during the transition from telogen to anagen. In response to glucocorticoid signaling, Tregs produce TGF-β3, which supports HFSC proliferation. Tregs express Notch ligand Jag1, which promotes HFSC differentiation. The epidermal SC immune niche comprises Th17 cells, Tc17 cells, dermal γδ T cells, CD8^+^ Trm cells, and (in mouse skin) DETCs. Though the role of these cells in homeostatic epidermal turnover is unclear, Tc17 cell–derived IL-17A induces production of antimicrobial peptides by the intact epidermis. (*b*) Residing at the base of the crypt, Lgr5^+^ intestinal SCs differentiate upward into progenitors called transit-amplifying cells that further give rise to the differentiated cell types (including enterocytes, goblet cells, entero-endocrine cells, tuft cells, and Paneth cells). Treg-derived IL-10 has been shown to promote intestinal SC self-renewal in vitro. Homeostatic IL-17 signals from an undefined source(s) are sensed by IL-17RA on intestinal SCs and induce differentiation to secretory lineages via the transcription factors NF-κB and ATOH. Abbreviations: AMP, antimicrobial peptide; DETC, dendritic epidermal γδ T cell; HFSC, hair follicle SC; IEL, intraepithelial lymphocyte; ILC3, type 3 innate lymphoid cell; MAIT, mucosal-associated invariant T; SC, stem cell; Tc17, CD8^+^IL-17A^+^ T; TGF-β3, transforming growth factor β3; Th17, T helper type 17; Treg, regulatory T cell; Trm, resident memory T. Figure adapted from images created with BioRender.com.

The skin and gut epithelia are naturally self-renewing tissues. Human skin epithelium remarkably turns over every 42 days (8 to 10 days in mice) (14). Gut epithelial cells are replaced every 2–6 days in adult mammals (15, 16). Herein, we refer to this baseline epithelial turnover as homeostatic regeneration. Stem cells (SCs) and progenitor cells of the interfollicular epidermis reside in the (basal) layer of the epidermis, and SCs of the intestinal epithelia reside in intestinal crypts [intestinal SCs (ISCs)] and locally fuel homeostatic regeneration (17, 18) (Figure 1). As SCs differentiate into various lineages, they move upward to eventually be sloughed off the skin’s surface or into the intestinal lumen (Figure 1a,b). Specialized hair follicle SCs (HFSCs) undergo cyclical bouts of rest (telogen) and regeneration (anagen), which coincide with hair growth (19). SCs are highly attuned to their environment or niche and adjust their behavior in response to niche-derived signals. Immune cells have surfaced as dominant members of the SC niches in the skin and gut, particularly under duress (20). Emerging evidence also supports a role for homeostatic cross talk between immune and various epithelial SC populations (19).

HFSCs and the cycling hair follicle are an ideal system for studying immune-epithelial cross talk during homeostatic regeneration. Immune cells dynamically localize to the perifollicular region over the course of the natural hair cycle and exert their influence on HFSCs. In particular, two tissue-resident immune cell types, macrophages and Foxp3^+^ regulatory T cells (Tregs), have emerged as key instructors of HFSC behavior. Macrophages influence the hair follicle cycle in a number of distinct ways. During telogen, a subset of perifollicular TREM2^+^ macrophages accumulate around the resting HF bulge. These TREM2^+^ macrophages secrete oncostatin M to maintain HFSC quiescence and restrain the hair cycle (21). The transition from telogen to anagen is facilitated by dying macrophages that release Wnt ligands to induce HFSC activation (22). In addition, macrophages also serve as an essential source of iron for HFSCs. Specific depletion of an iron exporter, ferroportin, disrupts the hair cycle, leading to hair loss (23). Whether these diverse functions are carried out by distinct subsets of macrophages or whether the same population of perifollicular macrophages dynamically change their behavior over the course of the hair cycle is unclear.

Treg regulation of HFSCs is evident after hair follicles are coaxed into cycling by hair plucking or depilation, an injury-like state commonly used to probe mechanisms of HFSC activation and differentiation. Following depilation, perifollicular Tregs provide essential Jag1 signals and induce Notch-mediated HFSC differentiation (24). More recently, glucocorticoid signaling was shown to collaborate with the canonical Treg transcription factor Foxp3 to induce TGF-β (transforming growth factor beta) production and HFSC proliferation (25). Whether and how Tregs contribute to the natural hair follicle cycle is still unclear. Additionally, under duress, hair follicles express a number of different chemokines to summon immune cells to their vicinity (26, 27). Whether resting and active follicles express distinct chemokines to control the composition of the perifollicular immune milieu during homeostatic regeneration remains to be tested.

In the intestine and skin, homeostatic lymphocytes constitutively express IL-17 in response to colonizing microbiota (28, 29). Systematic deletion of an IL-17 receptor (IL-17RA) on distinct epithelial cells revealed a critical role for IL-17 signaling in promoting differentiation of secretory cells from Lgr5^+^ ISCs and bolstering epithelial structure (30). On the other hand, IL-10 from in vitro–generated Tregs preserves stemness by promoting ISC self-renewal in intestinal organoids through a yet undefined mechanism (31). IL-10-deficient animals are particularly susceptible to experimental colitis, and single-nucleotide polymorphisms at the IL-10 locus are associated with early-onset colitis, suggesting that homeostatic IL-10 signaling may be essential for maintaining barrier function (32, 33). In contrast to the intestine, however, the role of immune-derived signals in fueling the homeostatic turnover of the interfollicular epidermis is unexplored. IL-17A from skin-dwelling CD8^+^IL-17A^+^ T cells (Tc17) cells drives the expression of antimicrobial peptides in the intact epidermis (20). Thus, it is tempting to speculate that epidermal turnover may be dynamically regulated by resident immune cells. SCs robustly express many cytokine receptors and immunomodulatory factors; however, studies examining the roles of these factors have largely been performed using organoid models, which lack tissue context (31). Similar to the cell-specific IL-17R depletion strategies employed by Lin et al. (30), probing the role of IL-10R (and other cytokine receptors) in skin and gut epithelial SCs at steady state is sure to yield insight into the dynamic regulation of barrier tissue fitness and function in health.

## TISSUE REPAIR: NATURE’S LESS THAN PERFECT SOLUTION

Many vertebrates and invertebrates possess the extraordinary ability to regenerate functional organs even after injury. These organisms are able to perfectly restore tissue architecture and functionality to their preinjury state (34). By contrast, mammals heal primarily via repair, a process that differs vastly from homeostatic regeneration. This largely imperfect response relies on rapid and haphazard deposition of extracellular matrix (ECM) to plug the damage, and it results in the generation of a nonfunctioning mass of fibrotic tissue known as a scar (35). Scarring repair may have arisen from an evolutionary “need for speed” to restore barrier function and protect from dangers looming in the terrestrial environment. Supporting this notion, early in gestation, humans and other mammals can perfectly regenerate certain tissues (36). Embryonic skin transitions from scarless healing during the first two trimesters to scar formation late in gestation (37). Importantly, however, not all fetal tissues engage regenerative responses, as early gestational wounds of the gastrointestinal tract form scars (38).

Inflammation is a key distinguishing feature of the fetal regenerative and adult repair responses. In contrast to adult wounds, for instance, fetal wounds do not elicit neutrophils or other proinflammatory mediators (39). Inoculating fetal wounds with bacteria induced inflammation and diverted responses to adult-like fibrotic repair, suggesting that microbial and other environmental triggers divert responses from regeneration toward repair (40). Paradoxically, studies in immune development and cell mapping efforts have noted the presence of immune cells across many fetal tissues, including skin and gut tissues (41, 42). Developing in the yolk sac, macrophages dominate the gestational immune milieu (43). In highly regenerative species, like the salamander, depletion of macrophages early after limb amputation hinders regrowth and results in the formation of a fibrotic stump (44). The role of fetal macrophages in regeneration after wounding has yet to be examined. These immune cells are known to engage in cross talk with distinct tissue components and perform developmental functions including neuronal synaptic pruning in the brain, endothelial connections in the kidney, and lymphatic patterning in the heart (45-47). Thus, it is tempting to speculate that developmental macrophages may uniquely promote fetal regeneration. Decoding the regenerative powers of developmental macrophages and the mechanisms of their cross talk with developing tissue could open the door to harnessing macrophage-based regenerative therapies throughout life.

## PHASES OF TISSUE REPAIR

As discussed above, mammalian tissue repair is a rapid process typified by inflammation and fibrosis. Repair occurs in a series of stages that are common to all wounds but regulated by different cell types and factors based on the type of damage and organ involved. Physiological tissue repair is classically divided into four distinct yet overlapping phases: hemostasis, inflammation, proliferation, and remodeling (34). Failure to engage these responses results in a range of nonhealing conditions or reparative pathologies, which we discuss in the section titled Immune-Mediated Epithelial Pathologies of Repair. Importantly, while inflammation is described as a separate phase of repair, burgeoning evidence supports a role for immune cells in nearly every facet of healing.

Hemostasis, the first phase, occurs immediately after traumatic damage, leading to platelet activation, a fibrin mesh, and the formation of a blood clot over the wound area. This sequence of events ceases blood flow and prevents excessive red blood cell loss from the circulation and produces a provisional scaffold for cells in a wound bed (48).

During the inflammatory phase, damaged tissues place an emergency call to alert the body by releasing danger-associated molecular patterns (DAMPs), reactive oxygen species, and alarmins (49). In addition, barrier organs such as the skin and gut organs must also cope with translocating microbes and their by-products or pathogen-associated molecular patterns (PAMPs). Collectively, these signals recruit neutrophils and monocytes from circulation and usher in the inflammatory phase of repair. Here it is important to note that the skin and the gut house a myriad of resident immune cells capable of sensing early danger signals emitted by tissue damage (13). Although the relative contribution of damaged epithelium versus resident immune cells to sensing early molecular signatures of damage and initiating the inflammatory phase is unclear, it is highly probable that optimal repair demands cooperation of these two cellular compartments. Wound inflammation is critical to prevent infection, clear debris, and supply growth factors and other signals that facilitate the proliferative phase of repair.

The proliferative phase involves a massive expansion of various cell types in both the epidermis and dermis to generate new tissue. During the proliferative phase, epithelial healing occurs in a process called re-epithelialization, which involves the proliferation of epithelial cells at the wound’s edge and their subsequent migration into the wound bed (50). Simultaneously the underlying mesenchymal cells of the dermis or lamina propria generate new connective or granulation tissue by fibroblast proliferation and differentiation and deposition of fibrotic ECM. Human wounds heal predominantly via re-epithelialization. By contrast, in mice and other mammals with loose skin that does not adhere to underlying structures, wound repair predominantly involves contraction mediated by the panniculus carnosus (51). To supply this newly generated tissue with oxygen and nutrients, optimal repair requires neovascularization, which occurs primarily through angiogenesis.

As the newly formed tissue matures, the remodeling phase of repair ensues to restore homeostasis. Apoptosis slows the massive expansion of epithelial, endothelial, fibroblast, immune, and other cells in the wound bed (52). How do tissues know when to stop growing and how much to reduce their size? The precise triggers and mediators of this large-scale reduction are poorly understood. However, decreased cellularity and excess collagen deposition drive the evolution of granulation tissue into a scar, ultimately compromising the tissue’s architecture and function (53).

While the stages of repair are universal, rebuilding each organ requires consideration of its unique cellular constituents, structure, and function. The skin and gut epithelia both house resident microbes. The skin, however, is lined by a multilayered stratified squamous epithelium, while the gut comprises a single layer of columnar epithelia with many specialized cell types that are extensively reviewed in Reference 19 (Figure 1). Below we discuss how resident and recruited immune cells help reconstruct the epithelial barrier following injury.

## IMMUNE SIGNALS IN RE-EPITHELIALIZATION

**Figure 2:**
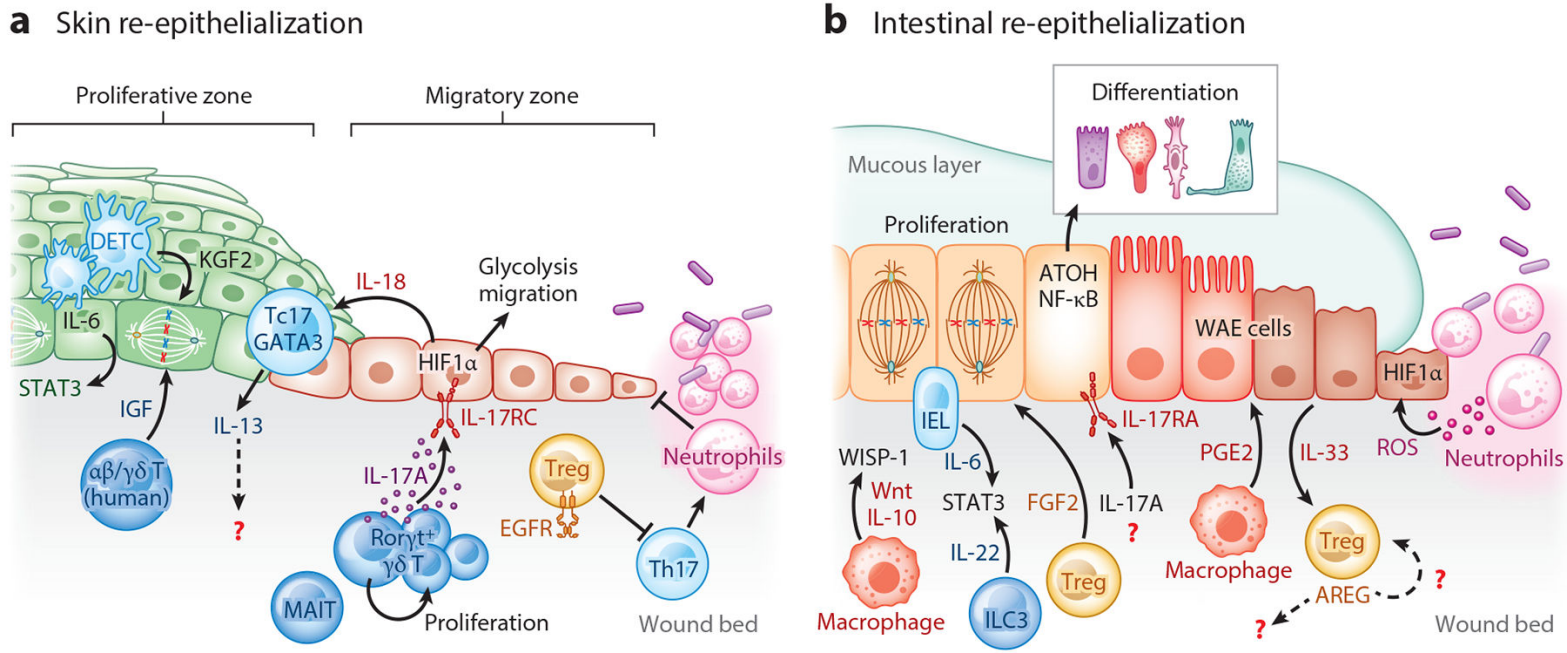
Immune-epithelial cross talk drives re-epithelialization of the (*a*) skin and (*b*) gut following damage. (*a*) Skin re-epithelization in proliferative and migratory zones. In the mouse skin, DETCs sustain epithelial proliferation by producing KGF2 and IL-6, which induces STAT3. In humans, resident αβ and γδ T cells produce IGF. Damaged epithelia secrete the alarmin IL-18, which signals resident Tc17 cells to express the transcription factor GATA3 and produce IL-13. Tregs require EGFR signaling to limit aberrant inflammation. Wound Tregs curb Th17 responses that promote neutrophils and impair healing. Resident tissue lymphocytes proliferate at the site of injury. Along with MAIT cells, RORγt^+^ γδ T cells produce IL-17A that induces epithelial HIF1α to drive a program of glycolysis to fuel migration. (*b*) Following intestinal injury, IL-6 from IELs and IL-22 from ILC3s induce STAT3 activation and epithelial proliferation. Macrophages release mitogenic Wnts and IL-10, which stimulate intestinal stem cell proliferation by inducing WISP-1. Tregs produce FGF2, which in concert with IL-17A drives proliferation of intestinal epithelial cells. Signaling via IL-17RA, a receptor for IL-17A, also induces epithelial differentiation to secretory lineages by inducing expression of the transcription factors NF-κB and ATOH. IL-33 released from damaged epithelium stimulates Treg amphiregulin production. Macrophage-derived prostaglandin E_2_ induces differentiation of wound-associated epithelial cells, which migrate to seal the breach. Neutrophils produce ROS, which induces HIF1α. Abbreviations: AREG, amphiregulin; DETC, dendritic epidermal γδ T cell; EGFR, epidermal growth factor receptor; FGF2, fibroblast growth factor 2; HIF1α, hypoxia-inducible factor 1 α; IEL, intraepithelial lymphocyte; IGF, insulin-like growth factor; ILC3, group 3 innate lymphoid cell; KGF2, keratinocyte growth factor; MAIT, mucosal-associated invariant T; PGE2, prostaglandin E_2_; ROS, reactive oxygen species; Tc17, CD8^+^IL-17A^+^ T; Th17, T helper 17; Treg, regulatory T cell; WAE, wound-associated epithelium; WISP-1, WNT1-inducible signaling protein 1. Figure adapted from images created with BioRender.com.

To limit the penetration of harmful agents, restitution of the epithelial barrier following erosion is of paramount importance in the skin and gut. As noted above, this is achieved through a process called re-epithelialization, which involves two key steps: (*a*) epithelial proliferation to expand cellularity for new tissue and (*b*) migration of epithelial cells to seal the breach. Remarkably these epithelial responses are spatially segregated into distinct compartments often referred to as the proliferative zone and the migratory zone, separated by a transition zone (Figure 2a). Thus, distinct molecular programs are sequentially engaged to induce epithelial proliferation and then migration, and wound immune cells are increasingly recognized as being involved in inducing these programs.

### Immune Cells Supply Proliferative Factors

In homeostasis, SC self-renewal and proliferation are dynamically regulated by gradients of Wnt, Notch, BMP, and Hippo signals from their local niche (reviewed in References 18 and 54). β-Catenin signaling induced by Wnt ligands is especially critical for SC proliferation (55). At steady state, both mesenchymal niche cells and the epithelium itself produce Wnt ligands to fuel regeneration. Macrophages have been identified as critical sources of mitogenic Wnt ligands after injury (56). In vitro studies first identified Wnt1 expression in macrophages isolated from ulcerative colitis patients (57). Macrophage-specific deletion of porcupine *O*-acyltransferase, an integral component of the Wnt secretion machinery, rendered mice vulnerable to radiation-induced intestinal injury (58). This failure to heal was traced to loss of ISCs and was only evident following damage. In addition to directly producing Wnts, macrophage-derived IL-10 also promotes proliferation by stimulating WNT1-inducible signaling protein 1 expression in ISCs (59). Macrophage-derived TNF-α can also induce β-catenin to prompt HFSC proliferation in a Wnt-independent manner, revealing a convergence of inflammatory and developmental signaling in repair (60).

Pioneering studies by Havran and colleagues first revealed that, in addition to immunosurveillance, epidermal resident lymphocytes also participate in repair (61). The very first evidence that lymphocytes were a potent source of epithelial mitogens came from studies coculturing dendritic epidermal γδ T cells (DETCs) with keratinocytes. DETCs produced keratinocyte growth factor 2, which was capable of promoting epithelial proliferation in vitro (55). This pro-healing function was also observed in vivo. Mice globally lacking γδ T cells had a severe reduction in the wound’s proliferative response. Analogous production of insulin-like growth factor (IGF) was observed in human skin-resident αβ and γδ T cells (62). Since these early studies, our understanding of lymphocyte populations has vastly expanded and the mechanisms by which they facilitate repair have been illuminated.

In contrast to pathogen responses that take upwards of a week to evoke antigen-specific T cells, injury responses are rapid and engage preexisting tissue lymphocytes. Many of these homeostatic populations are solicited by commensal microbes and rapidly proliferate at the site of injury (63). Indeed, systematic mapping of repair-associated lymphocytes revealed an expansion of innate lymphoid cells (64), mucosal-associated invariant T cells, γδ T cells, and Tregs as early as three days after injury (65). Moreover, these tissue-resident cells were sufficient for re-epithelialization, as treating mice with FTY720, which blunts migration of circulating lymphocytes, did not alter epithelial healing.

Resident lymphocytes are exquisitely positioned to integrate signals from damaged epithelium to induce pro-repair factors. In dextran sodium sulfate (DSS)-induced colitis, TGF-β from damaged epithelium triggered fibroblast growth factor 2 (FGF2) production in Tregs. In combination with IL-17A produced by Th17, FGF2 promoted epithelial proliferation (66). IL-17RA signaling can also synergize with epidermal growth factor receptor (EGFR) to stimulate epithelial proliferation (67). The precise signaling mechanisms of such cooperation between inflammatory and growth factors remain elusive. For instance, EGFR dimerization enables its activation and signal transduction (68). Similarly, IL-17RA requires the SFER domain of IL-17RC in order to signal (69). The mechanisms by which the IL-17RA and EGFR interact and signal to promote proliferation of epithelial cells thus warrant further study.

Damaged epithelia robustly secrete alarmins such as IL-18 and IL-33. IL-18 signaling in epidermal resident Tc17 cells rapidly induces expression of the transcription factor GATA3 and the cytokine IL-13 (70). Similarly, epithelial IL-33 activates the STR2 receptor on Tregs and stimulates production of amphiregulin (AREG), a potent EGFR ligand (71-74). In addition to providing growth factors, wound Tregs must also sustain their canonical immunoregulatory function to limit further damage. Indeed, Treg-derived AREG limits inflammation in muscle repair (75). Intriguingly, Tregs themselves express EGFR, and Treg-specific *Egfr* deletion results in heighted IFN-γ and inflammatory macrophages in skin wounds, suggesting that AREG could autonomously regulate Treg function in wounds (76). This anti-inflammatory role of Tregs is not absolute, as recent reports by Rosenblum and colleagues revealed that TGF-β from Tregs early after *Staphylococcus aureus* infection signals into epithelium to recruit neutrophils (77). Microbe-fighting neutrophils stall epithelial repair until the pathogen is cleared.

The transcription factor STAT3 has emerged as a central regulator of injury-induced epithelial proliferation. In some cases, STAT3 can entirely compensate for β-catenin signals and independently stimulate SC proliferation (78). IL-6 and IL-22 are key upstream inducers of STAT3 following injury. Acute IL-6 from intraepithelial lymphocytes instigates epithelial proliferation (79). In fact, delayed healing in old mice was traced to a failure of aged DETCs to produce IL-6 (80). Conversely, overexpression of the active form of an IL-6 receptor, gp130, drives epithelial proliferation (81). Surprisingly, however, hyperactive gp130 induces the Hippo pathway by triggering the transcription factor YAP to control cell growth independent of STAT3. Whether the magnitude or duration of gp130 activation results in divergent signaling and transcriptional effectors remains to be seen. Additionally, examining whether and how other STAT3-inducing factors, including IL-19, IL-20, and IL-24, modulate to epithelial proliferation during repair could yield insights into context-specific activators of this critical process (82).

Type 3 innate lymphoid cells (ILC3s) of the intestinal lamina propria are a critical source of IL-22, which concomitantly induces ISC proliferation and antimicrobial production in differentiated epithelial cells (78, 83-85). ILC3s are also capable of inducing ISC proliferation independent of IL-22/STAT3 by activating the Hippo-YAP pathway (86). Notably, ILC function in repair has largely been studied in the absence of adaptive immunity in mice, and may represent a compensatory mechanism or early response that can also be fulfilled by other innate-like lymphocytes. Given the paramount importance of repair to organismal survival, building cellular redundancy into the lymphocyte-epithelial cross talk may thus represent a cautionary feature of multicellular repair (87).

### Immune Cells Fuel Epithelial Differentiation and Migration

The contribution of epithelial SC proliferation to repair largely depends on the magnitude of damage, as smaller wounds are able to heal without cellular expansion. Repair, in this case, occurs through a process called epithelial restitution that relies entirely on differentiation and migration of epithelial cells at the wound’s edge (88, 89). Unlike immune cells that are highly mobile, epithelial cells are adherent and thus move via collective migration (90). That is, they maintain continuous attachment to their neighbors and move as a group, rather than individually migrating into the wound bed. In cutaneous wounds, a contiguous group of migrating epithelial cells is called a migrating tongue, and in intestinal wounds these cells are commonly referred to as wound-associated epithelial cells (87, 91) (Figure 2). Molecular characterization of migrating epithelial cells has revealed that these specialized repair cells are distinct from steady-state SCs and their differentiated progeny. Mobilized epithelial cells are enriched for signatures of epithelial to mesenchymal transition, hypoxia, and inflammation (87).

Disrupted vasculature from tissue damage results in oxygen deprivation in wounds. In addition, infiltrating neutrophils potentiate a hypoxic environment by competing for molecular O_2_ and producing hypoxia-promoting reactive oxygen species (92). Hypoxia-inducible factors (HIFs) are highly conserved transcription factors that mediate cellular adaptation to low-oxygen microenvironments (93). Stabilizing HIF1α in migrating epithelial cells is vital for epithelial restoration in both skin and gut repair (94, 95). Moreover, augmenting HIF1α in nonhealing diabetic wounds kick-starts repair (96). In addition to regulating genes involved in metabolic adaptation, HIF1α enhances the epithelial expression of intestinal trefoil factor 3, to bolster epithelial barrier function and facilitate repair upon damage (97), underscoring the central role of this transcription factor as a master regulator of repair-associated epithelial differentiation and migration.

Owing to its name, HIF1α in wounds was largely attributed to hypoxia. Yet, single-cell sequencing studies identified coexpression of inflammatory and hypoxia-responsive signatures in epithelial migrating tongues, raising the intriguing possibility that these two processes are interconnected (87). Indeed, we found that loss of dermal RORγt^+^ γδ T cells or epithelial-specific loss of IL-17RC impaired formation of the migrating tongue (65). Surprisingly, levels of hypoxia were comparable in wild-type mice and those lacking RORγt cells. Supplying exogenous IL-17A rescued the re-epithelialization and HIF1α defects in RORγt-deficient animals. Thus immune-derived secondary signals are necessary for sustaining HIF1α-induced migratory programs and could be productively leveraged to drive repair in nonhealing wounds.

The epithelial edges of nonhealing wounds appear stuck in a perpetual proliferative cycle, unable to activate migratory programs (50). Paradoxically, in nonhealing wounds, epithelial cells robustly express MHC-II and CCL20 and illicit IL-17A-producing immune cells (98, 99). Why, then, do these wounds not heal? One possibility is that too much of a good thing may backfire. Indeed, in a mouse model of injury, depletion of Tregs leads to exuberant T helper 17 (Th17) responses, inducing CXLC5-mediated neutrophilia and consequently impairing repair (100). Here it is also important to note that Th17 cells, unlike tissue-resident Tregs and innate-like lymphocytes, expand late in the repair process (65). Indeed, nonhealing diabetic wounds are typified by persistent neutrophils, and their substrates obstruct re-epithelialization (101). This is in contrast to physiological healing, where first-responder neutrophils whose job it is to control microbes do not linger throughout repair. Thus, the same immune cells and signals that kick-start healing early promote nonhealing states when unchecked. Defining the molecular and immunological signals that usher wounds through phases of repair thus remains an open challenge in tackling nonhealing wounds.

## IMMUNE-TISSUE INTERACTIONS FACILITATE RE-EPITHELIALIZATION

The skin and gut are multilayered barriers in which epithelia are supported by mesenchymal cells (fibroblasts, adipocytes) that define tissue topology through ECM production and provide insulation (102); vascular and lymphatic endothelia that deliver nutrients, oxygen, and immune cells; a myriad of neurons that perform critical sensory functions (103); and other components. As such, each of these distinct cellular compartments must be rebuilt following damage to ensure tissue functionality, and each of these systems communicates with and receives instructive cues from immune cells.

### Mesenchyme-Immune Interactions

Neutrophils and macrophages are the predominant wound-associated immune cell populations. Neutrophils have recently been appreciated for their surprising role in regulating fibroblasts and ECM in several ways. They robustly produce growth factors that influence fibroblasts, endothelial cells, and macrophages (104). In addition, fibroblast interactions with neutrophils, even transiently, induced TGF-β1 and consequently ECM production (105). In addition to ECM produced by dermal fibroblasts in the wound edge, fibroblasts from the underlying fascia plug skin wounds by dragging the ECM as well as surrounding vessels, immune cells, and nerves upward (106). Neutrophils interact with and transfer wound matrices via integrin AM and β2 (107). How do neutrophils perform such diverse functions in wounds? Neutrophil heterogeneity is increasingly evident in health and disease (108). Wound neutrophils may either develop with distinct functionalities or adopt these features in response to signals in the local wound microenvironment. For instance, heat shock factor produced in wounds triggers neutrophil- and integrin-mediated matrix transfer (107). Given the multifaceted roles of neutrophils, one area begging for clarity is precisely how developmental versus wound signals contribute to neutrophil functional heterogeneity.

For nearly a century, the intimate interactions between macrophages and fibroblasts have been a subject of fascination and a canvas for the discovery of key cellular survival and growth factors (109). These interactions are particularly evident in wounds and evolve over the course of repair. Lucas et al. (110) used a temporal depletion strategy to examine the stage-specific contribution of macrophages to repair. Early ablation after wounding profoundly impaired granulation tissue formation, vascularization, and re-epithelialization. Midstage deletion resulted in wound hemorrhaging, and late depletion did not affect the repair response or scarring.

Arising from myofibroblast progenitors, adipocytes are critical components of the dermal mesenchyme that participate in both homeostatic regeneration of the epithelium and tissue repair (102). CD301b^+^ macrophage-derived IGF and platelet-derived growth factor C (PDGFC) induce proliferation of adipose precursors and facilitate healing. The adipose-macrophage cross talk is a two-way street, as inhibiting lipolysis compromises wound macrophage function and derails repair (111).

### Endothelial-Immune Interactions

Restoration of vascular architecture in wounds occurs via angiogenesis and depends critically on immune-derived signals (112, 113). Surprisingly, wound macrophages, not epithelial cells, are the predominant source of vascular endothelial growth factor A (VEGFA) (110, 114). Proangiogenic macrophages appear to be transcriptionally distinct from inflammatory or repair macrophages and arise from circulating CCR2^+^Ly6C^+^ monocytes (115). Live imaging of angiogenesis in murine and zebrafish wounds revealed that neutrophils are only transiently drawn to the tips of damaged vessels, whereas macrophages are persistently associated with vessels and direct remodeling and regression (116). The role of the adaptive immune system in angiogenesis is well documented in the context of myocardial repair (117-119); however, it is unclear whether and how the adaptive immune system contributes to wound angiogenesis at epithelial barriers. Though formally untested in repair, T cells have been shown to secrete VEGFA in vitro and influence the behavior of macrophages via IFN-γ (120, 121).

In addition to vasculature, lymphatic vessels are also disrupted in wounds (122). Physical damage of lymphatic vessels can lead to accumulation of interstitial fluid, and restoration of tissue homeostasis requires recovery of lymphatic drainage through vessel regeneration. Lymphedema profoundly delays wound healing, suggesting that proper drainage of interstitial fluids is vital for healing (123). Wound lymphangiogenesis is stimulated by VEGFC or VEGFD signaling and occurs in parallel with vascular angiogenesis (124). Recruited macrophages and activated platelets produce VEGFC and VEGFD in wounds (125-128). However, how other immune cells and released mediators support lymphatic regeneration during tissue repair remains an open question. In addition, lymphatic endothelial cells produce paracrine signals that regulate homeostatic intestinal and skin SC behavior (129, 130), cardiac growth (131), and thermogenesis of brown adipose tissue (132), raising the possibility that lymphatics may also be a source of growth factors during re-epithelialization.

### Neuro-Immune Interactions

The skin and gut barriers are densely innervated with many types of neurons that mediate sensation and relay information to the brain. We now appreciate that in addition to canonical sensory function neurons engage in bidirectional communication with immune cells to facilitate repair (133-135). Sensory neurons robustly express receptors for inflammatory cytokines including IL-1R, IL-17RA, IL-6R, IL-4R, and TNFR, resulting in pain or itch signaling upon inflammation (136). Immune cells in turn express receptors for neuropeptides and transmitters such as dopamine, substance P, and neuropeptides such as calcitonin gene–related peptide (CGRP) (137). Illustrative of this cross talk, ligation of the neuroregulatory receptor RET in intestinal ILCs triggers IL-22 production (138). During intestinal worm infection, IFN-γ-activated enteric glia release CXCL10, resulting in the regulation of granulomas (139). Close interactions between neurons and macrophages regulate barrier integrity and intestinal physiology, but how these interactions contribute to repair is an open question (140, 141). In the skin, however, exchanges between the nervous and immune systems are vital to recover from sunburn-induced injury. TAFA4 from mechanosensory neurons induces macrophage IL-10 and limits inflammation (142). Physical parameters such as pressure or touch in wounds may be essential in activating such mechanosensory neurons, just as noxious agents trigger nociceptors. Recently, microbial metabolites have been shown to drive repair after sciatic nerve damage. Indole-6-phosphate from intestinal microbiota promotes axonal regeneration and epidermal innervation. This response is mediated by neutrophil chemotaxis to the nerve bodies in the dorsal root ganglion, where they presumably supply regenerative factors (143).

There is much yet to be discovered about multisystem repair. The aforementioned interactions between immune cells and the tissue parenchyma only scratch the surface. Systematically charting the myriad of interactions as tissues are rebuilt may require us to zoom out and start unbiasedly tracking tissue responses. Moreover, it is unclear whether repair engages the same mechanisms as development when tissues are first built, or whether the rules of rebuilding tissues are entirely rewritten with age.

## IMMUNE-MEDIATED EPITHELIAL PATHOLOGIES OF REPAIR

Given their importance to organismal survival, repair responses have been reinforced with a high level of molecular and cellular redundancy (34). However, a number of diseases arise from either a failure to launch repair programs (e.g., nonhealing wounds and cancers) or exuberant repair (e.g., epithelial inflammatory diseases). Unsurprisingly, the immune system has a hand in driving these pathologies, and the same players identified in the repair process often underlie epithelial diseases. Below we focus on epithelial cancers and two inflammatory conditions, psoriasis and inflammatory bowel disease (IBD), which are wound-like states that are excessively healed or undergo cycles of injury and repair, respectively (144).

### Cancer: Wounds That Do Not Heal

In 1863 Rudolf Virchow proposed his chronic irritation theory, concluding that irritation and subsequent inflammation lead to formation of neoplastic tissues (145). Over a century later, Harold Dvorak observed the tumor stroma and famously referred to tumors as “wounds that do not heal” (144). A wealth of data identifying shared molecular and cellular features of wounds and tumors now support Dvorak’s notion that tumors are stuck in a form of persistent damage and accordingly are chronically inflamed (146).

Early observations of links between inflammation and cancer were made when chickens infected with the Rous sarcoma virus developed tumors when wounded (147). These observations in tumorigenesis have since been traced to the same inflammatory pathways (IL-1, IL-6, IL-17, and IL-22) that underlie repair (148-153). Studies using chemically induced skin carcinogenesis have pinpointed a requirement for IL-17A signaling in cancer cells to drive tumor growth (67). Not only are immune factors shared between wounds and tumors, but these two processes also engage the same SCs. Lineage tracing revealed that Lrig1^+^ SCs that direct repair in response to IL-17 signaling also constitute the majority of tumor mass (67). In the intestine, IL-17 signaling similarly enhances the proliferation and survival of enterocytes with a mutation in the tumor suppressor *APC* gene, contributing to adenoma formation (152). A growing body of evidence also supports a role for IL-22 from Th17 cells and ILC3s in tumor development (153-155). IL-22 enhances cancer stemness and tumorigenic potential in colorectal cancer by promoting STAT3 activation and expression of the histone 3 lysine 79 (H3K79) methyltransferase DOT1L (153). Thus, immune signals that instigate repair programs are also evident in the tumor microenvironment and reinforce unchecked proliferation caused by tumor mutations that enable cancer cells to overcome cell-cycle checkpoints.

### Epithelial Inflammatory Diseases: Psoriasis and Inflammatory Bowel Disease

Psoriasis and IBD are prototypic chronic remitting and relapsing inflammatory diseases of the skin and gut, respectively (156). Though the pathologies of these two diseases are driven by similar inflammatory cytokines, IL-17A, IL-22, and TNFα, they arise from very different manifestations of repair. Psoriatic pathology involves epidermal hyperthickening, hypervascularization, innervation, and aberrant mesenchymal response, which are reminiscent of an amplified repair response, or “over healing” (157-159). By contrast, in inflammatory bowel diseases, and in particular Crohn disease, pathology is mediated by repeated cycles of epithelial injury and repair, or “recurrent healing” (160, 161). These contrasting repair pathologies also provide insight into therapy responsiveness or lack there of in the two diseases. IL-17 blockade has been lauded for its success in psoriasis, likely due to restraint of the epithelial hyperproliferative and differentiation pathology (162, 163). In other words, blocking the inflammatory responses in psoriasis patients limits over healing. By contrast, in IBD where IL-17 signals may be crucial to boost epithelial repair and cope with recurrent injury, biologics that target this cytokine have exacerbated disease symptoms (164). Thus, defining the immune milieu in inflammatory epithelial diseases is likely not sufficient to determine optimal interventions, as these factors may be either causal in driving disease or consequential in coping with pathology. Instead, understanding the impact of immune factors on tissue function and repair will help inform rational and lasting therapies.

## CONCLUSIONS AND PERSPECTIVE

As scientists eavesdrop on the conversations between immune and epithelial cells, they overhear increasingly intricate discussions in a variety of contexts. In homeostasis, immune cells that reside in the skin and gut not only surveil the tissue for interlopers but also act as local sources of growth factors to sustain the epithelium. This convergence of immune and regenerative programs may represent a cost-saving measure on the part of the host, as bolstering the physical epithelial barrier may be more energetically efficacious than mounting repeated inflammatory responses to penetrating pathogens. The evolutionary alliance between immune cells and the epithelium strengthens further following injury. Immune signals amplify epithelial cell functions (proliferation, differentiation, and migration) to expedite repair, and these same features are co-opted pathologically by inflammatory diseases and cancers.

A burgeoning area in the context of immune-epithelial communications and repair is that of inflammatory memory. We and others have found that epithelial SCs of the skin and gut maintain a memory of their inflammatory encounters that fundamentally alter their tissue repair functions (165, 166). Memory of inflammation in SCs is encoded at the level of chromatin, by maintaining accessibility and histone modifications at key stress-responsive loci (167). These memory domains are bookmarked by both general stress-responsive transcription factors like FOS-JUN and inflammatory transcription factors like STAT3. Indeed, in the intestine, ablating epithelial IL-6 was sufficient to abrogate the memory response (165). In addition, epithelial memory of inflammation was also responsible for setting the inflammatory tone of the intestine and controlling the numbers of homeostatic Th17 cells. However, studies with repeated limb amputations in highly regenerative axolotls or following intestinal damage in flies and mice have revealed that there are limits to the reparative boost provided by inflammatory memory (168, 169). The immunological factors underlying these reparative roadblocks over time and experience require clarification. Nevertheless, it is tempting to speculate that accumulating inflammatory factors over time, reminiscent of inflammaging, tip the reparative scales away from healing (170). Indeed, aging is associated with profound defects in epithelial repair, which have at least in part been attributed to a breakdown of normal immune-epithelial cross talk.

Targeting immune-epithelial communication to boost repair or mitigate inflammatory pathologies represents a new frontier in the treatment of inflammatory diseases. In this regard, synthetic immunology is emerging as an exciting new discipline to modulate the function of immune mediators. Saxton and colleagues (171) exemplified the power of manipulating cytokine structure to obtain defined outcomes. They developed synthetic IL-22 agonists that preserved the tissue-regenerative function of this cytokine by inducing STAT3 signaling without involving any of the inflammatory factors induced by STAT1. Another exciting application of synthetic immunology may be the use of immune cell therapies in repair. CAR (chimeric antigen receptor) T cell therapies that localize to and kill tumors have revolutionized cancer therapy. Recently, this same technology was used to target fibrosis following cardiac injury (172). In addition, engineering cells to supply growth or antimicrobial factors in hard-to-treat and infected wounds could help deliver highly localized payloads. Epithelial SC–derived tissue engraftments have been clinically and/or experimentally successful in the skin and the gut to treat epithelial genetic disorders or inflammatory conditions that compromise barrier function in isolated cases (173). Yet, the scalability of such approaches remains limited, in part because engraftment in the context of inflammation is challenging. Thus, reprogramming the inflammatory tone of tissues and priming them for engraftment by dampening proinflammatory factors and augmenting pro-repair factors could transform SC-based repair modalities.

Immune-epithelial cross talk stands out as an exemplar of multicellularity and the systems of cooperation that ensure rapid repair. Decoding the fascinating dialogue not just in the skin and gut epithelia but in all epithelial cells of the body is sure to reveal their unique and universal features. Finding unique tissue-specific repair mechanisms may enable the development of focal repair therapies, while universal reparative programs could unravel systemic therapies. Defining the immune contexture and functionality of repair is both a century-old question and an exciting new frontier in immunology and regenerative medicine.

## Figures and Tables

**Table 1:** Comprehensive list of mediators of immune-epithelial cross talk in regeneration

Mediator/signaling pathway	Source	Target	Function	Reference
Iron	Skin macrophages	HFSCs	Activation and hair cycle	23
Oncostatin M	Skin TREM2^+^ macrophages	HFSCs	Sustains quiescence state	21
Wnts	Skin macrophages	HFSCs	Activation and hair cycle	22
Notch signaling through Jag1	Skin Tregs	HFSCs	Differentiation and proliferation	22
Glucocorticoid receptor–TGF-β3 axis	Skin Tregs	HFSCs	Instructs perifollicular Tregs to produce TGF-β3 and prompt HFSC differentiation	25
IL-17A	Gut Th17 cells	Lgr5^+^ ISCs	Promotes secretory cell lineage commitment	30, 31
IL-10	Gut Tregs	ISCs	Supports ISC renewal in intestinal organoids	31

Abbreviations: HFSC, hair follicle stem cell; ISC, intestinal stem cell; Treg, regulatory T cell.

**Table 2:** Comprehensive list of mediators of immune-epithelial cross talk in repair

Mediator/signaling pathway	Source	Target	Function	Reference
**Epithelial-immune interactions**				
Wnt ligands, Wnt1	Gut macrophages	ISCs	Repair after injury	55-57
IL-10	Gut macrophages	ISCs	Triggers expression of WISP-1 in ISCs to promote proliferation	59
FGF2	Gut Tregs	Epithelial cells	During colitis, microbiota-driven TGF-β1 controls FGF2 production in Tregs. FGF2 cooperates with IL-17 to promote repair of damaged intestinal epithelium	66
IL-33	Gut epithelial cells	Tregs	Stimulates production of amphiregulin	71-74
IL-6	Gut intraepithelial lymphocytes	Intestinal epithelial progenitors	Epithelial proliferation and wound repair	79
IL-22	Gut ILC3s	ISCs	STAT3 activation and β-catenin-independent proliferation	79, 83-85
Hippo-YAP1 induction	Gut ILC3s	ISCs	Induction of Hippo-YAP1 pathway independently of IL-22/STAT3 to preserve ISCs following acute damage	86
KGF2	DETCs	Keratinocytes	Proliferation in vitro	55
TNF-α	Skin macrophages	HFSCs	Wnt-independent β-catenin activation and HFSC proliferation	60
IL-17A	?	Lrig1^+^ stem cells	IL-17R recruits EGFR for IL-17A signaling in Lrig1^+^ cells to induce their proliferation during wound healing and wound-induced tumorigenesis	67
IL-18	Skin epithelial cells	Tc17 cells	IL-18 from wounded epithelium signals epidermal resident Tc17 cells to induce GATA3 expression and produce IL-13	70
IL-17A	Skin RORγt^+^ γδ T cells	Wound-edge epithelial cells	IL-17A supplied by RORγt^+^ γδ T cells is necessary for optimal HIF1α activation in the wound-edge epithelium. The IL-17A–HIF1α axis directs the metabolic rewiring of damaged epithelium toward a program of glycolysis to fuel migration	65
**Mesenchyme-immune interactions**				
IGF and PDGFC	CD301b^+^ macrophages	Adipose precursors	Drive proliferation of adipose precursors and facilitate healing	111
**Endothelial-immune interactions**				
VEGF-A	Macrophages	Endothelial cells	Promotes angiogenesis during repair	110, 114
VEGF-C and VEGF-D	Macrophages/platelets	Lymphatic endothelial cells	Promote lymphangiogenesis during repair	124-128
**Neuro-immune interactions**				
Neurotrophic factors	Enteric neuroglia	ILC3s	Ligation to the neuroregulatory receptor RET triggers the production of IL-22	138
TAFA4	Skin mechanosensory receptors	Macrophages	Induces macrophage IL-10 and limits wound inflammation	142

Abbreviations: DETC, dendritic epidermal γδ T cell; FGF2, fibroblast growth factor 2; HFSC, hair follicle stem cell; IGF, insulin-like growth factor; ILC3, type 3 innate lymphoid cell; ISC, intestinal stem cell; KGF2, keratinocyte growth factor 2; PDGFC, platelet-derived growth factor C; Tc17, CD8^+^IL-17A^+^ T cell; Treg, regulatory T cell; VEGF-A, vascular endothelial growth factor A.

## References

[1] Melms JC, Biermann J, Huang H, Wang Y, Nair A, 2021. A molecular single-cell lung atlas of lethal COVID-19. Nature 595(7865):114–19 33915568 10.1038/s41586-021-03569-1 PMC8814825

[2] Delorey TM, Ziegler CGK, Heimberg G, Normand R, Yang Y, 2021. COVID-19 tissue atlases reveal SARS-CoV-2 pathology and cellular targets. Nature 595(7865):107–13 33915569 10.1038/s41586-021-03570-8 PMC8919505

[3] Wynn T. 2008. Cellular and molecular mechanisms of fibrosis. J. Pathol 214(2):199–210 18161745 10.1002/path.2277 PMC2693329

[4] Bootun R. 2013. Effects of immunosuppressive therapy on wound healing. Int. Wound J 10(1):98–104 22364410 10.1111/j.1742-481X.2012.00950.x PMC7950386

[5] Metchnikoff E. 1894. The comparative pathology of inflammation, transl. FA Starling, EH Starling. Nature 50(1287):194–95 (from French)

[6] Ebert RH, Florey HW. 1939. The extravascular development of the monocyte observed in vivo. Br. J. Exp. Pathol 20(4):342–56

[7] Leibovich SJ, Ross R. 1975. The role of the macrophage in wound repair: a study with hydrocortisone and antimacrophage serum. Am. J. Pathol 78(1):71–100 1109560 PMC1915032

[8] Polverini PJ, Cotran RS, Gimbrone MA, Unanue ER. 1977. Activated macrophages induce vascular proliferation. Nature 269(5631):804–6 927505 10.1038/269804a0

[9] Hunt TK, Knighton DR, Thakral KK, Goodson WH, Andrews WS. 1984. Studies on inflammation and wound healing: angiogenesis and collagen synthesis stimulated in vivo by resident and activated wound macrophages. Surgery 96(1):48–54 6204395

[10] Murray PJ, Allen JE, Biswas SK, Fisher EA, Gilroy DW, 2014. Macrophage activation and polarization: nomenclature and experimental guidelines. Immunity 41(1):14–20 25035950 10.1016/j.immuni.2014.06.008 PMC4123412

[11] Wynn TA, Vannella KM. 2016. Macrophages in tissue repair, regeneration, and fibrosis. Immunity 44(3):450–62 26982353 10.1016/j.immuni.2016.02.015 PMC4794754

[12] Gordon S, Plüddemann A. 2017. Tissue macrophages: heterogeneity and functions. BMC Biol. 15(1):53 28662662 10.1186/s12915-017-0392-4 PMC5492929

[13] Belkaid Y, Harrison OJ. 2017. Homeostatic immunity and the microbiota. Immunity 46(4):562–76 28423337 10.1016/j.immuni.2017.04.008 PMC5604871

[14] Potten CS, Saffhill R, Maibach HI. 1987. Measurement of the transit time for cells through the epidermis and stratum corneum of the mouse and guinea-pig. Cell Prolif. 20(5):461–72 10.1111/j.1365-2184.1987.tb01355.x 3450396

[15] Williams JM, Duckworth CA, Burkitt MD, Watson AJM, Campbell BJ, Pritchard DM. 2015. Epithelial cell shedding and barrier function. Vet. Pathol 52(3):445–55 25428410 10.1177/0300985814559404 PMC4441880

[16] Creamer B, Shorter RG, Bamforth J. 1961. The turnover and shedding of epithelial cells. I. The turnover in the gastro-intestinal tract. Gut 2:110–18 13696345 10.1136/gut.2.2.110 PMC1413255

[17] Blanpain C, Fuchs E. 2006. Epidermal stem cells of the skin. Annu. Rev. Cell Dev. Biol 22:339–73 16824012 10.1146/annurev.cellbio.22.010305.104357 PMC2405915

[18] Gehart H, Clevers H. 2019. Tales from the crypt: new insights into intestinal stem cells. Nat. Rev. Gastroenterol. Hepatol 16(1):19–34 30429586 10.1038/s41575-018-0081-y

[19] Rosenblum D, Naik S. 2022. Epithelial-immune crosstalk in health and disease. Curr. Opin. Genet. Dev 74:101910 35461159 10.1016/j.gde.2022.101910 PMC9170062

[20] Naik S, Bouladoux N, Linehan JL, Han S-J, Harrison OJ, 2015. Commensal-dendritic-cell interaction specifies a unique protective skin immune signature. Nature 520(7545):104–8 25539086 10.1038/nature14052 PMC4667810

[21] Wang ECE, Dai Z, Ferrante AW, Drake CG, Christiano AM. 2019. A subset of TREM2^+^ dermal macrophages secretes oncostatin M to maintain hair follicle stem cell quiescence and inhibit hair growth. Cell Stem Cell 24(4):654–69.e6 30930146 10.1016/j.stem.2019.01.011

[22] Castellana D, Paus R, Perez-Moreno M. 2014. Macrophages contribute to the cyclic activation of adult hair follicle stem cells. PLOS Biol. 12(12):e1002002 25536657 10.1371/journal.pbio.1002002 PMC4275176

[23] Recalcati S, Gammella E, Buratti P, Doni A, Anselmo A, 2019. Macrophage ferroportin is essential for stromal cell proliferation in wound healing. Haematologica 104(1):47–58 30115660 10.3324/haematol.2018.197517 PMC6312033

[24] Ali N, Zirak B, Rodriguez RS, Pauli ML, Truong H-A, 2017. Regulatory T cells in skin facilitate epithelial stem cell differentiation. Cell 169(6):1119–29.e11 28552347 10.1016/j.cell.2017.05.002 PMC5504703

[25] Liu Z, Hu X, Liang Y, Yu J, Li H, 2022. Glucocorticoid signaling and regulatory T cells cooperate to maintain the hair-follicle stem-cell niche. Nat. Immunol. 23(7):1086–97 35739197 10.1038/s41590-022-01244-9 PMC9283297

[26] Nagao K, Kobayashi T, Moro K, Ohyama M, Adachi T, 2012. Stress-induced production of chemokines by hair follicles regulates the trafficking of dendritic cells in skin. Nat. Immunol 13(8):744–52 22729248 10.1038/ni.2353 PMC4115277

[27] Lay K, Yuan S, Gur-Cohen S, Miao Y, Han T, 2018. Stem cells repurpose proliferation to contain a breach in their niche barrier. eLife 7:e41661 30520726 10.7554/eLife.41661 PMC6324878

[28] Ivanov II, Atarashi K, Manel N, Brodie EL, Shima T, 2009. Induction of intestinal Th17 cells by segmented filamentous bacteria. Cell 139(3):485–98 19836068 10.1016/j.cell.2009.09.033 PMC2796826

[29] Naik S, Bouladoux N, Wilhelm C, Molloy MJ, Salcedo R, 2012. Compartmentalized control of skin immunity by resident commensals. Science 337(6098):1115–19 22837383 10.1126/science.1225152 PMC3513834

[30] Lin X, Gaudino SJ, Jang KK, Bahadur T, Singh A, 2022. IL-17RA-signaling in Lgr5^+^ intestinal stem cells induces expression of transcription factor ATOH1 to promote secretory cell lineage commitment. Immunity 55(2):237–53.e8 35081371 10.1016/j.immuni.2021.12.016 PMC8895883

[31] Biton M, Haber AL, Rogel N, Burgin G, Beyaz S, 2018. T helper cell cytokines modulate intestinal stem cell renewal and differentiation. Cell 175(5):1307–20.e22 30392957 10.1016/j.cell.2018.10.008 PMC6239889

[32] Gunasekera DC, Ma J, Vacharathit V, Shah P, Ramakrishnan A, 2020. The development of colitis in Il10^−/−^ mice is dependent on IL-22. Mucosal Immunol. 13(3):493–506 31932715 10.1038/s41385-019-0252-3 PMC7566780

[33] Moran CJ, Walters TD, Guo C-H, Kugathasan S, Klein C, 2013. IL-10R polymorphisms are associated with very early-onset ulcerative colitis. Inflamm. Bowel Dis 19(1):115–23 22550014 10.1002/ibd.22974 PMC3744177

[34] Gurtner GC, Werner S, Barrandon Y, Longaker MT. 2008. Wound repair and regeneration. Nature 453(7193):314–21 18480812 10.1038/nature07039

[35] Konieczny P, Naik S. 2021. Healing without scarring. Science 372(6540):346–47 33888629 10.1126/science.abi5770

[36] Rowlatt U. 1979. Intrauterine wound healing in a 20 week human fetus. Virchows Arch. A Path. Anat. Histol. 381(3):353–61 155931 10.1007/BF00432477

[37] Longaker MT, Chiu ES, Adzick NS, Stern M, Harrison MR, Stern R. 1991. Studies in fetal wound healing. V. A prolonged presence of hyaluronic acid characterizes fetal wound fluid. Ann. Surg 213(4):292–96 2009010 10.1097/00000658-199104000-00003 PMC1358347

[38] Mast BA, Albanese CT, Kapadia S. 1998. Tissue repair in the fetal intestinal tract occurs with adhesions, fibrosis, and neovascularization. Ann. Plast. Surg 41(2):140–44 9718146 10.1097/00000637-199808000-00005

[39] Larson BJ, Longaker MT, Lorenz HP. 2010. Scarless fetal wound healing: a basic science review. Plast. Reconstr. Surg 126(4):1172–80 20885241 10.1097/PRS.0b013e3181eae781 PMC4229131

[40] Frantz FW, Bettinger DA, Haynes JH, Johnson DE, Harvey KM, 1993. Biology of fetal repair: The presence of bacteria in fetal wounds induces an adult-like healing response. J. Pediatr. Surg 28(3):428–34 8468658 10.1016/0022-3468(93)90243-e

[41] Reynolds G, Vegh P, Fletcher J, Poyner EFM, Stephenson E, 2021. Developmental cell programs are co-opted in inflammatory skin disease. Science 371(6527):eaba6500 33479125 10.1126/science.aba6500 PMC7611557

[42] Elmentaite R, Kumasaka N, Roberts K, Fleming A, Dann E, 2021. Cells of the human intestinal tract mapped across space and time. Nature 597(7875):250–55 34497389 10.1038/s41586-021-03852-1 PMC8426186

[43] Lavin Y, Mortha A, Rahman A, Merad M. 2015. Regulation of macrophage development and function in peripheral tissues. Nat. Rev. Immunol 15(12):731–44 26603899 10.1038/nri3920 PMC4706379

[44] Godwin JW, Pinto AR, Rosenthal NA. 2013. Macrophages are required for adult salamander limb regeneration. PNAS 110(23):9415–20 23690624 10.1073/pnas.1300290110 PMC3677454

[45] Pasciuto E, Burton OT, Roca CP, Lagou V, Rajan WD, 2020. Microglia require CD4 T cells to complete the fetal-to-adult transition. Cell 182(3):625–40.e24 32702313 10.1016/j.cell.2020.06.026 PMC7427333

[46] Munro DA, Wineberg Y, Tarnick J, Vink CS, Li Z, 2019. Macrophages restrict the nephrogenic field and promote endothelial connections during kidney development. eLife 8:e43271 30758286 10.7554/eLife.43271 PMC6374076

[47] Cahill TJ, Sun X, Ravaud C, Villa del Campo C, Klaourakis K, 2021. Tissue-resident macrophages regulate lymphatic vessel growth and patterning in the developing heart. Development 148(3):dev194563 33462113 10.1242/dev.194563 PMC7875498

[48] Pool JG. 1977. Normal hemostatic mechanisms: a review. Am. J. Med. Technol 43(8):776–80 888856

[49] Wilgus TA. 2018. Alerting the body to tissue injury: the role of alarmins and DAMPs in cutaneous wound healing. Curr. Pathobiol. Rep 6(1):55–60. 29862143 10.1007/s40139-018-0162-1 PMC5978745

[50] Pastar I, Stojadinovic O, Yin NC, Ramirez H, Nusbaum AG, 2014. Epithelialization in wound healing: a comprehensive review. Adv. Wound Care 3(7):445–64 10.1089/wound.2013.0473 PMC4086220 25032064

[51] Galiano RD, Michaels JV, Dobryansky M, Levine JP, Gurtner GC. 2004. Quantitative and reproducible murine model of excisional wound healing. Wound Repair Regen. 12(4):485–92 15260814 10.1111/j.1067-1927.2004.12404.x

[52] Desmoulière A, Redard M, Darby I, Gabbiani G. 1995. Apoptosis mediates the decrease in cellularity during the transition between granulation tissue and scar. Am. J. Pathol 146(1):56–66 7856739 PMC1870783

[53] Lovvorn HN, Cheung DT, Nimni ME, Perelman N, Estes JM, Adzick NS. 1999. Relative distribution and crosslinking of collagen distinguish fetal from adult sheep wound repair. J. Pediatr. Surg 34(1):218–23 10022176 10.1016/s0022-3468(99)90261-0

[54] Hsu Y-C, Li L, Fuchs E. 2014. Emerging interactions between skin stem cells and their niches. Nat. Med 20(8):847–56 25100530 10.1038/nm.3643 PMC4358898

[55] Nelson WJ, Nusse R. 2004. Convergence of Wnt, β-catenin, and cadherin pathways. Science 303(5663):1483–87 15001769 10.1126/science.1094291 PMC3372896

[56] Vannella KM, Wynn TA. 2017. Mechanisms of organ injury and repair by macrophages. Annu. Rev. Physiol 79:593–617 27959618 10.1146/annurev-physiol-022516-034356

[57] Cosín-Roger J, Ortiz-Masiá D, Calatayud S, Hernández C, Álvarez A, 2013. M2 macrophages activate WNT signaling pathway in epithelial cells: relevance in ulcerative colitis. PLOS ONE 8(10):e78128 24167598 10.1371/journal.pone.0078128 PMC3805515

[58] Saha S, Aranda E, Hayakawa Y, Bhanja P, Atay S, 2016. Macrophage-derived extracellular vesicle-packaged WNTs rescue intestinal stem cells and enhance survival after radiation injury. Nat. Commun 7(1):13096 27734833 10.1038/ncomms13096 PMC5065628

[59] Quiros M, Nishio H, Neumann PA, Siuda D, Brazil JC, 2017. Macrophage-derived IL-10 mediates mucosal repair by epithelial WISP-1 signaling. J. Clin. Investig 127(9):3510–20 28783045 10.1172/JCI90229 PMC5669557

[60] Wang X, Chen H, Tian R, Zhang Y, Drutskaya MS, 2017. Macrophages induce AKT/β-catenin-dependent Lgr5^+^ stem cell activation and hair follicle regeneration through TNF. Nat. Commun 8(1):14091 28345588 10.1038/ncomms14091 PMC5378973

[61] Jameson J, Ugarte K, Chen N, Yachi P, Fuchs E, 2002. A role for skin γδ T cells in wound repair. Science 296(5568):747–49 11976459 10.1126/science.1069639

[62] Toulon A, Breton L, Taylor KR, Tenenhaus M, Bhavsar D, 2009. A role for human skin-resident T cells in wound healing. J. Exp. Med 206(4):743–50 19307328 10.1084/jem.20081787 PMC2715110

[63] Linehan JL, Harrison OJ, Han S-J, Byrd AL, Vujkovic-Cvijin I, 2018. Non-classical immunity controls microbiota impact on skin immunity and tissue repair. Cell 172(4):784–96.e18 29358051 10.1016/j.cell.2017.12.033 PMC6034182

[64] Vivier E, Artis D, Colonna M, Diefenbach A, Di Santo JP, 2018. Innate lymphoid cells: 10 years on. Cell 174(5):1054–66 30142344 10.1016/j.cell.2018.07.017

[65] Konieczny P, Xing Y, Sidhu I, Subudhi I, Mansfield KP, 2022. Interleukin-17 governs hypoxic adaptation of injured epithelium. Science 377(6602):eabg9302 35709248 10.1126/science.abg9302 PMC9753231

[66] Song X, Dai D, He X, Zhu S, Yao Y, 2015. Growth factor FGF2 cooperates with interleukin-17 to repair intestinal epithelial damage. Immunity 43(3):488–501 26320657 10.1016/j.immuni.2015.06.024

[67] Chen X, Cai G, Liu C, Zhao J, Gu C, 2019. IL-17R-EGFR axis links wound healing to tumorigenesis in Lrig1^+^ stem cells. J. Exp. Med 216(1):195–214 30578323 10.1084/jem.20171849 PMC6314525

[68] Wee P, Wang Z. 2017. Epidermal growth factor receptor cell proliferation signaling pathways. Cancers 9(5):52 28513565 10.3390/cancers9050052 PMC5447962

[69] Ho AW, Shen F, Conti HR, Patel N, Childs EE, 2010. IL-17RC is required for immune signaling via an extended SEF/IL-17R signaling domain in the cytoplasmic tail. J. Immunol 185(2):1063–70 20554964 10.4049/jimmunol.0903739 PMC2897912

[70] Harrison OJ, Linehan JL, Shih H-Y, Bouladoux N, Han S-J, 2019. Commensal-specific T cell plasticity promotes rapid tissue adaptation to injury. Science 363(6422):eaat6280 30523076 10.1126/science.aat6280 PMC7304459

[71] Molofsky AB, Savage AK, Locksley RM. 2015. Interleukin-33 in tissue homeostasis, injury, and inflammation. Immunity 42(6):1005–19 26084021 10.1016/j.immuni.2015.06.006 PMC4471869

[72] Lam AJ, MacDonald KN, Pesenacker AM, Juvet SC, Morishita KA, 2019. Innate control of tissue-reparative human regulatory T cells. J. Immunol 202(8):2195–209 30850479 10.4049/jimmunol.1801330

[73] Schiering C, Krausgruber T, Chomka A, Fröhlich A, Adelmann K, 2014. The alarmin IL-33 promotes regulatory T-cell function in the intestine. Nature 513(7519):564–68 25043027 10.1038/nature13577 PMC4339042

[74] Cosovanu C, Neumann C. 2020. The many functions of Foxp3^+^ regulatory T cells in the intestine. Front. Immunol 11:600973 33193456 10.3389/fimmu.2020.600973 PMC7606913

[75] Burzyn D, Kuswanto W, Kolodin D, Shadrach JL, Cerletti M, 2013. A special population of regulatory T cells potentiates muscle repair. Cell 155(6):1282–95 24315098 10.1016/j.cell.2013.10.054 PMC3894749

[76] Nosbaum A, Prevel N, Truong H-A, Mehta P, Ettinger M, 2016. Regulatory T cells facilitate cutaneous wound healing. J. Immunol 196(5):2010–14 26826250 10.4049/jimmunol.1502139 PMC4761457

[77] Moreau JM, Dhariwala MO, Gouirand V, Boda DP, Boothby IC, 2021. Regulatory T cells promote innate inflammation after skin barrier breach via TGF-β activation. Sci. Immunol 6(62):eabg2329 34452925 10.1126/sciimmunol.abg2329 PMC8958044

[78] Lindemans CA, Calafiore M, Mertelsmann AM, O’Connor MH, Dudakov JA, 2015. Interleukin-22 promotes intestinal-stem-cell-mediated epithelial regeneration. Nature 528(7583):560–64 26649819 10.1038/nature16460 PMC4720437

[79] Kuhn KA, Manieri NA, Liu T-C, Stappenbeck TS. 2014. IL-6 stimulates intestinal epithelial proliferation and repair after injury. PLOS ONE 9(12):e114195 25478789 10.1371/journal.pone.0114195 PMC4257684

[80] Keyes BE, Liu S, Asare A, Naik S, Levorse J, 2016. Impaired epidermal to dendritic T-cell signaling slows wound repair in aged skin. Cell 167(5):1323–38.e14 27863246 10.1016/j.cell.2016.10.052 PMC5364946

[81] Taniguchi K, Wu L-W, Grivennikov SI, de Jong PR, Lian I, 2015. A gp130-Src-YAP module links inflammation to epithelial regeneration. Nature 519(7541):57–62 25731159 10.1038/nature14228 PMC4447318

[82] Dudakov JA, Hanash AM, van den Brink MRM. 2015. Interleukin-22: immunobiology and pathology. Annu. Rev. Immunol 33:747–85 25706098 10.1146/annurev-immunol-032414-112123 PMC4407497

[83] Aparicio-Domingo P, Romera-Hernandez M, Karrich JJ, Cornelissen F, Papazian N, 2015. Type 3 innate lymphoid cells maintain intestinal epithelial stem cells after tissue damage. J. Exp. Med 212(11):1783–91 26392223 10.1084/jem.20150318 PMC4612094

[84] Pickert G, Neufert C, Leppkes M, Zheng Y, Wittkopf N, 2009. STAT3 links IL-22 signaling in intestinal epithelial cells to mucosal wound healing. J. Exp. Med 206(7):1465–72 19564350 10.1084/jem.20082683 PMC2715097

[85] Zheng Y, Valdez PA, Danilenko DM, Hu Y, Sa SM, 2008. Interleukin-22 mediates early host defense against attaching and effacing bacterial pathogens. Nat. Med 14(3):282–89 18264109 10.1038/nm1720

[86] Romera-Hernandez M, Aparicio-Domingo P, Papazian N, Karrich JJ, Cornelissen F, 2020. Yap1-driven intestinal repair is controlled by group 3 innate lymphoid cells. Cell Rep. 30(1):37–45.e3 31914395 10.1016/j.celrep.2019.11.115

[87] Haensel D, Jin S, Sun P, Cinco R, Dragan M, 2020. Defining epidermal basal cell states during skin homeostasis and wound healing using single-cell transcriptomics. Cell Rep. 30(11):3932–47.e6 32187560 10.1016/j.celrep.2020.02.091 PMC7218802

[88] Lacy ER. 1988. Epithelial restitution in the gastrointestinal tract. J. Clin. Gastroenterol 10(Suppl. 1):S72–77 3053884 10.1097/00004836-198812001-00012

[89] Aragona M, Dekoninck S, Rulands S, Lenglez S, Mascré G, 2017. Defining stem cell dynamics and migration during wound healing in mouse skin epidermis. Nat. Commun 8(1):14684 28248284 10.1038/ncomms14684 PMC5339881

[90] Friedl P, Gilmour D. 2009. Collective cell migration in morphogenesis, regeneration and cancer. Nat. Rev. Mol. Cell Biol 10(7):445–57 19546857 10.1038/nrm2720

[91] Miyoshi H, VanDussen KL, Malvin NP, Ryu SH, Wang Y, 2017. Prostaglandin E2 promotes intestinal repair through an adaptive cellular response of the epithelium. EMBO J. 36(1):5–24 27797821 10.15252/embj.201694660 PMC5210160

[92] Campbell EL, Bruyninckx WJ, Kelly CJ, Glover LE, McNamee EN, 2014. Transmigrating neutrophils shape the mucosal microenvironment through localized oxygen depletion to influence resolution of inflammation. Immunity 40(1):66–77 24412613 10.1016/j.immuni.2013.11.020 PMC3951457

[93] Semenza GL. 2012. Hypoxia-inducible factors in physiology and medicine. Cell 148(3):399–408 22304911 10.1016/j.cell.2012.01.021 PMC3437543

[94] Hong WX, Hu MS, Esquivel M, Liang GY, Rennert RC, 2014. The role of hypoxia-inducible factor in wound healing. Adv. Wound Care 3(5):390–99 10.1089/wound.2013.0520 PMC4005494 24804159

[95] Wang Y, Chiang I-L, Ohara TE, Fujii S, Cheng J, 2019. Long-term culture captures injury-repair cycles of colonic stem cells. Cell 179(5):1144–59.e15 31708126 10.1016/j.cell.2019.10.015 PMC6904908

[96] Botusan IR, Sunkari VG, Savu O, Catrina AI, Grünler J, 2008. Stabilization of HIF-1α is critical to improve wound healing in diabetic mice. PNAS 105(49):19426–31 19057015 10.1073/pnas.0805230105 PMC2614777

[97] Furuta GT, Turner JR, Taylor CT, Hershberg RM, Comerford K, 2001. Hypoxia-inducible factor 1-dependent induction of intestinal trefoil factor protects barrier function during hypoxia. J. Exp. Med 193(9):1027–34 11342587 10.1084/jem.193.9.1027 PMC2193432

[98] Liu Z, Zhang L, Toma MA, Li D, Bian X, 2022. Integrative small and long RNA omics analysis of human healing and nonhealing wounds discovers cooperating microRNAs as therapeutic targets. eLife 11:e80322 35942686 10.7554/eLife.80322 PMC9374442

[99] Li D, Cheng S, Pei Y, Sommar P, Karner J, 2022. Single-cell analysis reveals major histocompatibility complex II–expressing keratinocytes in pressure ulcers with worse healing outcomes. J. Investig. Dermatol 142(3 Part A):705–16 34536485 10.1016/j.jid.2021.07.176

[100] Mathur AN, Zirak B, Boothby IC, Tan M, Cohen JN, 2019. Treg-cell control of a CXCL5-IL-17 inflammatory axis promotes hair-follicle-stem-cell differentiation during skin-barrier repair. Immunity 50(3):655–67.e4 30893588 10.1016/j.immuni.2019.02.013 PMC6507428

[101] Wong SL, Demers M, Martinod K, Gallant M, Wang Y, 2015. Diabetes primes neutrophils to undergo NETosis, which impairs wound healing. Nat. Med 21(7):815–19 26076037 10.1038/nm.3887 PMC4631120

[102] Plikus MV, Wang X, Sinha S, Forte E, Thompson SM, 2021. Fibroblasts: origins, definitions, and functions in health and disease. Cell 184(15):3852–72 34297930 10.1016/j.cell.2021.06.024 PMC8566693

[103] Tamari M, Ver Heul AM, Kim BS. 2021. Immunosensation: neuroimmune cross talk in the skin. Annu. Rev. Immunol 39:369–93 33561366 10.1146/annurev-immunol-101719-113805

[104] Theilgaard-Mönch K, Knudsen S, Follin P, Borregaard N. 2004. The transcriptional activation program of human neutrophils in skin lesions supports their important role in wound healing. J. Immunol 172(12):7684–93 15187151 10.4049/jimmunol.172.12.7684

[105] Curaj A, Schumacher D, Rusu M, Staudt M, Li X, 2020. Neutrophils modulate fibroblast function and promote healing and scar formation after murine myocardial infarction. Int. J. Mol. Sci 21(10):3685 32456225 10.3390/ijms21103685 PMC7279328

[106] Correa-Gallegos D, Jiang D, Christ S, Ramesh P, Ye H, 2019. Patch repair of deep wounds by mobilized fascia. Nature 576(7786):287–92 31776510 10.1038/s41586-019-1794-y

[107] Fischer A, Wannemacher J, Christ S, Koopmans T, Kadri S, 2022. Neutrophils direct preexisting matrix to initiate repair in damaged tissues. Nat. Immunol 23(4):518–31 35354953 10.1038/s41590-022-01166-6 PMC8986538

[108] Ng LG, Ostuni R, Hidalgo A. 2019. Heterogeneity of neutrophils. Nat. Rev. Immunol 19(4):255–65 30816340 10.1038/s41577-019-0141-8

[109] Buechler MB, Fu W, Turley SJ. 2021. Fibroblast-macrophage reciprocal interactions in health, fibrosis, and cancer. Immunity 54(5):903–15 33979587 10.1016/j.immuni.2021.04.021

[110] Lucas T, Waisman A, Ranjan R, Roes J, Krieg T, 2010. Differential roles of macrophages in diverse phases of skin repair. J. Immunol 184(7):3964–77 20176743 10.4049/jimmunol.0903356

[111] Shook BA, Wasko RR, Mano O, Rutenberg-Schoenberg M, Rudolph MC, 2020. Dermal adipocyte lipolysis and myofibroblast conversion are required for efficient skin repair. Cell Stem Cell 26(6):880–95.e6 32302523 10.1016/j.stem.2020.03.013 PMC7853423

[112] Ribatti D, Crivellato E. 2009. Immune cells and angiogenesis. J. Cell. Mol. Med 13(9a):2822–33 19538473 10.1111/j.1582-4934.2009.00810.x PMC4498938

[113] Frantz S, Vincent KA, Feron O, Kelly RA. 2005. Innate immunity and angiogenesis. Circ. Res 96(1):15–26 15637304 10.1161/01.RES.0000153188.68898.ac

[114] Stockmann C, Kirmse S, Helfrich I, Weidemann A, Takeda N, 2011. A wound size-dependent effect of myeloid cell-derived vascular endothelial growth factor on wound healing. J. Investig. Dermatol 131(3):797–801 10.1038/jid.2010.345 21107350

[115] Willenborg S, Lucas T, van Loo G, Knipper JA, Krieg T, 2012. CCR2 recruits an inflammatory macrophage subpopulation critical for angiogenesis in tissue repair. Blood 120(3):613–25 22577176 10.1182/blood-2012-01-403386

[116] Gurevich DB, Severn CE, Twomey C, Greenhough A, Cash J, 2018. Live imaging of wound angiogenesis reveals macrophage orchestrated vessel sprouting and regression. EMBO J. 37(13):e97786 29866703 10.15252/embj.201797786 PMC6028026

[117] Kwee BJ, Budina E, Najibi AJ, Mooney DJ. 2018. CD4 T-cells regulate angiogenesis and myogenesis. Biomaterials 178:109–21 29920403 10.1016/j.biomaterials.2018.06.003 PMC6090550

[118] Hata T, Takahashi M, Hida S, Kawaguchi M, Kashima Y, 2011. Critical role of Th17 cells in inflammation and neovascularization after ischaemia. Cardiovasc. Res 90(2):364–72 21156823 10.1093/cvr/cvq397

[119] Weirather J, Hofmann UDW, Beyersdorf N, Ramos GC, Vogel B, 2014. Foxp3^+^ CD4^+^ T cells improve healing after myocardial infarction by modulating monocyte/macrophage differentiation. Circ. Res 115(1):55–67 24786398 10.1161/CIRCRESAHA.115.303895

[120] Mor F, Quintana FJ, Cohen IR. 2004. Angiogenesis-inflammation cross-talk: vascular endothelial growth factor is secreted by activated T cells and induces Th1 polarization. J. Immunol 172(7):4618–23 15034080 10.4049/jimmunol.172.7.4618

[121] Ishida Y, Kondo T, Takayasu T, Iwakura Y, Mukaida N. 2004. The essential involvement of cross-talk between IFN-γ and TGF-β in the skin wound-healing process. J. Immunol 172(3):1848–55 14734769 10.4049/jimmunol.172.3.1848

[122] Oliver G, Kipnis J, Randolph GJ, Harvey NL. 2020. The lymphatic vasculature in the 21st century: novel functional roles in homeostasis and disease. Cell 182(2):270–96 32707093 10.1016/j.cell.2020.06.039 PMC7392116

[123] Mallon EC, Ryan TJ. 1994. Lymphedema and wound healing. Clin. Dermatol 12(1):89–93 8180949 10.1016/0738-081x(94)90260-7

[124] Saaristo A, Tammela T, Fārkkilā A, Kärkkäinen M, Suominen E, 2006. Vascular endothelial growth factor-C accelerates diabetic wound healing. Am. J. Pathol 169(3):1080–87 16936280 10.2353/ajpath.2006.051251 PMC1698814

[125] Karpanen T, Alitalo K. 2008. Molecular biology and pathology of lymphangiogenesis. Annu. Rev. Pathol 3:367–97 18039141 10.1146/annurev.pathmechdis.3.121806.151515

[126] Lim L, Bui H, Farrelly O, Yang J, Li L, 2019. Hemostasis stimulates lymphangiogenesis through release and activation of VEGFC. Blood 134(20):1764–75 31562136 10.1182/blood.2019001736 PMC6856989

[127] Kataru RP, Jung K, Jang C, Yang H, Schwendener RA, 2009. Critical role of CD11b^+^ macrophages and VEGF in inflammatory lymphangiogenesis, antigen clearance, and inflammation resolution. Blood 113(22):5650–59 19346498 10.1182/blood-2008-09-176776

[128] Hadrian K, Willenborg S, Bock F, Cursiefen C, Eming SA, Hos D. 2021. Macrophage-mediated tissue vascularization: similarities and differences between cornea and skin. Front. Immunol 12:667830 33897716 10.3389/fimmu.2021.667830 PMC8058454

[129] Gur-Cohen S, Yang H, Baksh SC, Miao Y, Levorse J, 2019. Stem cell-driven lymphatic remodeling coordinates tissue regeneration. Science 366(6470):1218–25 31672914 10.1126/science.aay4509 PMC6996853

[130] Niec RE, Chu T, Schernthanner M, Gur-Cohen S, Hidalgo L, 2022. Lymphatics act as a signaling hub to regulate intestinal stem cell activity. Cell Stem Cell 29(7):1067–82.e18 35728595 10.1016/j.stem.2022.05.007 PMC9271639

[131] Liu X, De la Cruz E, Gu X, Balint L, Oxendine-Burns M, 2020. Lymphoangiocrine signals promote cardiac growth and repair. Nature 588(7839):705–11 33299187 10.1038/s41586-020-2998-x PMC7770123

[132] Li J, Li E, Czepielewski RS, Chi J, Guo X, 2021. Neurotensin is an anti-thermogenic peptide produced by lymphatic endothelial cells. Cell Metab. 33(7):1449–65.e6 34038712 10.1016/j.cmet.2021.04.019 PMC8266750

[133] Chu C, Artis D, Chiu IM. 2020. Neuro-immune interactions in the tissues. Immunity 52(3):464–74 32187517 10.1016/j.immuni.2020.02.017 PMC10710744

[134] Klein Wolterink RGJ, Wu GS, Chiu IM, Veiga-Fernandes H. 2022. Neuroimmune interactions in peripheral organs. Annu. Rev. Neurosci 45:339–60 35363534 10.1146/annurev-neuro-111020-105359 PMC9436268

[135] Matheis F, Muller PA, Graves CL, Gabanyi I, Kerner ZJ, 2020. Adrenergic signaling in muscularis macrophages limits infection-induced neuronal loss. Cell 180(1):64–78.e16 31923400 10.1016/j.cell.2019.12.002 PMC7271821

[136] Pinho-Ribeiro FA, Verri WA, Chiu IM. 2017. Nociceptor sensory neuron-immune interactions in pain and inflammation. Trends Immunol. 38(1):5–19 27793571 10.1016/j.it.2016.10.001 PMC5205568

[137] Veiga-Fernandes H, Mucida D. 2016. Neuro-immune interactions at barrier surfaces. Cell 165(4):801–11 27153494 10.1016/j.cell.2016.04.041 PMC4871617

[138] Ibiza S, García-Cassani B, Ribeiro H, Carvalho T, Almeida L, 2016. Glial-cell-derived neuroregulators control type 3 innate lymphoid cells and gut defence. Nature 535(7612):440–43 27409807 10.1038/nature18644 PMC4962913

[139] Progatzky F, Shapiro M, Chng SH, Garcia-Cassani B, Classon CH, 2021. Regulation of intestinal immunity and tissue repair by enteric glia. Nature 599(7883):125–30 34671159 10.1038/s41586-021-04006-z PMC7612231

[140] Gabanyi I, Muller PA, Feighery L, Oliveira TY, Costa-Pinto FA, Mucida D. 2016. Neuro-immune interactions drive tissue programming in intestinal macrophages. Cell 164(3):378–91 26777404 10.1016/j.cell.2015.12.023 PMC4733406

[141] Matteoli G, Gomez-Pinilla PJ, Nemethova A, Giovangiulio MD, Cailotto C, 2014. A distinct vagal anti-inflammatory pathway modulates intestinal muscularis resident macrophages independent of the spleen. Gut 63(6):938–48 23929694 10.1136/gutjnl-2013-304676

[142] Hoeffel G, Debroas G, Roger A, Rossignol R, Gouilly J, 2021. Sensory neuron-derived TAFA4 promotes macrophage tissue repair functions. Nature 594(7861):94–99 34012116 10.1038/s41586-021-03563-7

[143] Serger E, Luengo-Gutierrez L, Chadwick JS, Kong G, Zhou L, 2022. The gut metabolite indole-3 propionate promotes nerve regeneration and repair. Nature 607(7919):585–92 35732737 10.1038/s41586-022-04884-x

[144] Dvorak HF. 1986. Tumors: wounds that do not heal. N. Engl. J. Med 315(26):1650–59 3537791 10.1056/NEJM198612253152606

[145] Virchow R. 1863. Aetiologie der neoplastischen Geschwulste/Pathogenie der neoplastischen Geschwulste. Berlin: Verlag von August Hirschwald

[146] Schäfer M, Werner S. 2008. Cancer as an overhealing wound: an old hypothesis revisited. Nat. Rev. Mol. Cell Biol 9(8):628–38 18628784 10.1038/nrm2455

[147] Dolberg DS, Hollingsworth R, Hertle M, Bissell MJ. 1985. Wounding and its role in RSV-mediated tumor formation. Science 230(4726):676–78 2996144 10.1126/science.2996144

[148] Arwert EN, Lal R, Quist S, Rosewell I, van Rooijen N, Watt FM. 2010. Tumor formation initiated by nondividing epidermal cells via an inflammatory infiltrate. PNAS 107(46):19903–8 21041641 10.1073/pnas.1007404107 PMC2993377

[149] Schafer ZT, Brugge JS. 2007. IL-6 involvement in epithelial cancers. J. Clin. Investig 117(12):3660–63 18060028 10.1172/JCI34237 PMC2096452

[150] Cataisson C, Salcedo R, Michalowski AM, Klosterman M, Naik S, 2019. T-cell deletion of MyD88 connects IL17 and IκBζ to RAS oncogenesis. Mol. Cancer Res 17(8):1759–73 31164412 10.1158/1541-7786.MCR-19-0227 PMC6720116

[151] Grivennikov S, Karin E, Terzic J, Mucida D, Yu G-Y, 2009. IL-6 and Stat3 are required for survival of intestinal epithelial cells and development of colitis-associated cancer. Cancer Cell 15(2):103–13 19185845 10.1016/j.ccr.2009.01.001 PMC2667107

[152] Grivennikov SI, Wang K, Mucida D, Stewart CA, Schnabl B, 2012. Adenoma-linked barrier defects and microbial products drive IL-23/IL-17-mediated tumour growth. Nature 491(7423):254–58 23034650 10.1038/nature11465 PMC3601659

[153] Kryczek I, Lin Y, Nagarsheth N, Peng D, Zhao L, 2014. IL-22^+^CD4^+^ T cells promote colorectal cancer stemness via STAT3 transcription factor activation and induction of the methyltransferase DOT1L. Immunity 40(5):772–84 24816405 10.1016/j.immuni.2014.03.010 PMC4032366

[154] Bernshtein B, Curato C, Ioannou M, Thaiss CA, Gross-Vered M, 2019. IL-23-producing IL-10Rα-deficient gut macrophages elicit an IL-22-driven proinflammatory epithelial cell response. Sci. Immunol 4(36):eaau6571 31201258 10.1126/sciimmunol.aau6571 PMC6697185

[155] Kirchberger S, Royston DJ, Boulard O, Thornton E, Franchini F, 2013. Innate lymphoid cells sustain colon cancer through production of interleukin-22 in a mouse model. J. Exp. Med 210(5):917–31 23589566 10.1084/jem.20122308 PMC3646494

[156] Lowes MA, Suárez-Fariñas M, Krueger JG. 2014. Immunology of psoriasis. Annu. Rev. Immunol 32:227–55 24655295 10.1146/annurev-immunol-032713-120225 PMC4229247

[157] Morhenn VB. 1988. Keratinocyte proliferation in wound healing and skin diseases. Immunol. Today 9(4):104–7 2476150 10.1016/0167-5699(88)91278-9

[158] Mansbridge JN, Knapp AM. 1987. Changes in keratinocyte maturation during wound healing. J. Investig. Dermatol 89(3):253–63 2442269 10.1111/1523-1747.ep12471216

[159] Nickoloff BJ, Bonish BK, Marble DJ, Schriedel KA, DiPietro LA, 2006. Lessons learned from psoriatic plaques concerning mechanisms of tissue repair, remodeling, and inflammation. J. Investig. Dermatol. Symp. Proc 11(1):16–29 10.1038/sj.jidsymp.5650010 17069007

[160] de Souza HSP, Fiocchi C. 2016. Immunopathogenesis of IBD: current state of the art. Nat. Rev. Gastroenterol. Hepatol 13(1):13–27 26627550 10.1038/nrgastro.2015.186

[161] Rieder F, Brenmoehl J, Leeb S, Schölmerich J, Rogler G. 2007. Wound healing and fibrosis in intestinal disease. Gut 56(1):130–39 17172588 10.1136/gut.2006.090456 PMC1856649

[162] Langley RG, Elewski BE, Lebwohl M, Reich K, Griffiths CEM, 2014. Secukinumab in plaque psoriasis—results of two phase 3 trials. N. Engl. J. Med 371(4):326–38 25007392 10.1056/NEJMoa1314258

[163] Hawkes JE, Yan BY, Chan TC, Krueger JG. 2018. Discovery of the IL-23/IL-17 signaling pathway and the treatment of psoriasis. J. Immunol 201(6):1605–13 30181299 10.4049/jimmunol.1800013 PMC6129988

[164] Fauny M, Moulin D, D’Amico F, Netter P, Petitpain N, 2020. Paradoxical gastrointestinal effects of interleukin-17 blockers. Ann. Rheum. Dis 79(9):1132–38 32719044 10.1136/annrheumdis-2020-217927

[165] Lim AI, McFadden T, Link VM, Han S-J, Karlsson R-M, 2021. Prenatal maternal infection promotes tissue-specific immunity and inflammation in offspring. Science 373(6558):eabf3002 34446580 10.1126/science.abf3002

[166] Naik S, Larsen SB, Gomez NC, Alaverdyan K, Sendoel A, 2017. Inflammatory memory sensitizes skin epithelial stem cells to tissue damage. Nature 550(7677):475–80 29045388 10.1038/nature24271 PMC5808576

[167] Larsen SB, Cowley CJ, Sajjath SM, Barrows D, Yang Y, 2021. Establishment, maintenance, and recall of inflammatory memory. Cell Stem Cell 28(10):1758–74.e8 34320411 10.1016/j.stem.2021.07.001 PMC8500942

[168] Bryant DM, Sousounis K, Payzin-Dogru D, Bryant S, Sandoval AGW, 2017. Identification of regenerative roadblocks via repeat deployment of limb regeneration in axolotls. npj Regen. Med 2:30 29302364 10.1038/s41536-017-0034-z PMC5677943

[169] Haller S, Kapuria S, Riley RR, O’Leary MN, Schreiber KH, 2017. mTORC1 activation during repeated regeneration impairs somatic stem cell maintenance. Cell Stem Cell 21(6):806–18.e5 29220665 10.1016/j.stem.2017.11.008 PMC5823264

[170] Franceschi C, Garagnani P, Parini P, Giuliani C, Santoro A. 2018. Inflammaging: a new immune-metabolic viewpoint for age-related diseases. Nat. Rev. Endocrinol 14(10):576–90 30046148 10.1038/s41574-018-0059-4

[171] Saxton RA, Henneberg LT, Calafiore M, Su L, Jude KM, 2021. The tissue protective functions of interleukin-22 can be decoupled from pro-inflammatory actions through structure-based design. Immunity 54(4):660–72.e9 33852830 10.1016/j.immuni.2021.03.008 PMC8054646

[172] Rurik JG, Tombácz I, Yadegari A, Méndez Fernández PO, Shewale SV, 2022. CART cells produced in vivo to treat cardiac injury. Science 375(6576):91–96 34990237 10.1126/science.abm0594 PMC9983611

[173] Yui S, Nakamura T, Sato T, Nemoto Y, Mizutani T, 2012. Functional engraftment of colon epithelium expanded in vitro from a single adult Lgr5^+^ stem cell. Nat. Med 18(4):618–23 22406745 10.1038/nm.2695

